# Supporting At-Risk Youth and Their Families to Manage and Prevent Diabetes: Developing a National Partnership of Medical Residency Programs and High Schools

**DOI:** 10.1371/journal.pone.0158477

**Published:** 2016-07-06

**Authors:** Liana Gefter, Nancy Morioka-Douglas, Ashini Srivastava, Eunice Rodriguez

**Affiliations:** 1 Center for Primary Care Research, Division of General Medical Disciplines, Department of Medicine, Stanford University School of Medicine, Stanford, CA, United States of America; 2 Department of Pediatrics, Stanford University School of Medicine, Stanford, CA, United States of America; Vanderbilt University, UNITED STATES

## Abstract

**Background:**

The Stanford Youth Diabetes Coaches Program (SYDCP) is a school based health program in which Family Medicine residents train healthy at-risk adolescents to become diabetes self-management coaches for family members with diabetes. This study evaluates the impact of the SYDCP when disseminated to remote sites. Additionally, this study aims to assess perceived benefit of enhanced curriculum.

**Methods:**

From 2012–2015, 10 high schools and one summer camp in the US and Canada and five residency programs were selected to participate. Physicians and other health providers implemented the SYDCP with racial/ethnic-minority students from low-income communities. Student coaches completed pre- and posttest surveys which included knowledge, health behavior, and psychosocial asset questions (i.e., worth and resilience), as well as open-ended feedback questions. T-test pre-post comparisons were used to determine differences in knowledge and psychosocial assets, and open and axial coding methods were used to analyze qualitative data.

**Results:**

A total of 216 participating high school students completed both pre-and posttests, and 96 nonparticipating students also completed pre- and posttests. Student coaches improved from pre- to posttest significantly on knowledge (p<0.005 in 2012–13, 2014 camp, and 2014–15); worth (p<0.1 in 2014–15); problem solving (p<0.005 in 2014 camp and p<0.1 in 2014–15); and self-efficacy (p<0.05 in 2014 camp). Eighty-two percent of student coaches reported that they considered making a behavior change to improve their own health as a result of program participation. Qualitative feedback themes included acknowledgment of usefulness and relevance of the program, appreciation for physician instructors, knowledge gain, pride in helping family members, improved relationships and connectedness with family members, and lifestyle improvements.

**Conclusion:**

Overall, when disseminated, this program can increase health knowledge and some psychosocial assets of at-risk youth and holds promise to empower these youth with health literacy and encourage them to adopt healthy behaviors.

## Introduction

The burden of chronic disease in the US has reached epic proportions and disproportionately affects individuals from low-income, ethnic minority populations.[1] The impact extends to youth as evidenced by the prevalence of Type 2 Diabetes and prediabetes more than doubling among US adolescents in the last decade.[2] Ethnic minority communities historically have been disempowered and distrustful of the medical system.[3] Thus, providing opportunities for ethnic minority youth to become engaged with healthcare and empowered to improve their own health is particularly important.

One such opportunity is the Stanford Youth Diabetes Coaches Program (SYDCP), a school based health program that develops partnerships between high schools and Family Medicine residency programs (programs training medical school graduates for three years to become Family Medicine physicians). Family Medicine residents train healthy at-risk adolescents from low-income, ethnic minority communities to become diabetes self-management coaches for family members with diabetes. A local pilot study demonstrated that, compared to nonparticipants, student coaches showed significant improvements in knowledge, sense of belonging, and feelings of self-worth.[4] Additionally, the SYDCP had a positive impact on participating Family Medicine residents, increasing their intention to incorporate self-management support into their clinical practice and aiding in their acquisition of teaching skills; interpersonal and communication skills; and patient self-management support skills.[5]

In this paper we discuss the developmental phases and testing of the SYDCP. Developed using feedback from students, teachers, physician trainees and community members, the project maximizes interaction between physician trainees and students; emphasizes student and family voices; and incorporates technology to enhance engagement. We focus on evaluating the impact of the SYDCP when disseminated to remote sites in the US and Canada; as well as investigating whether the disseminated program can equip at-risk youth with knowledge and skills to enable them to become engaged in their own health and empowered to improve their own health behaviors. Additionally, this study aims to assess the benefits of enhanced curriculum that emphasizes discussion and interaction between physicians and students in class; as well as the perceived benefit of curriculum enhanced with technologies such as text reminders, emails, and embedded video content.

## Methods

### Program Design

**Fig 1:**
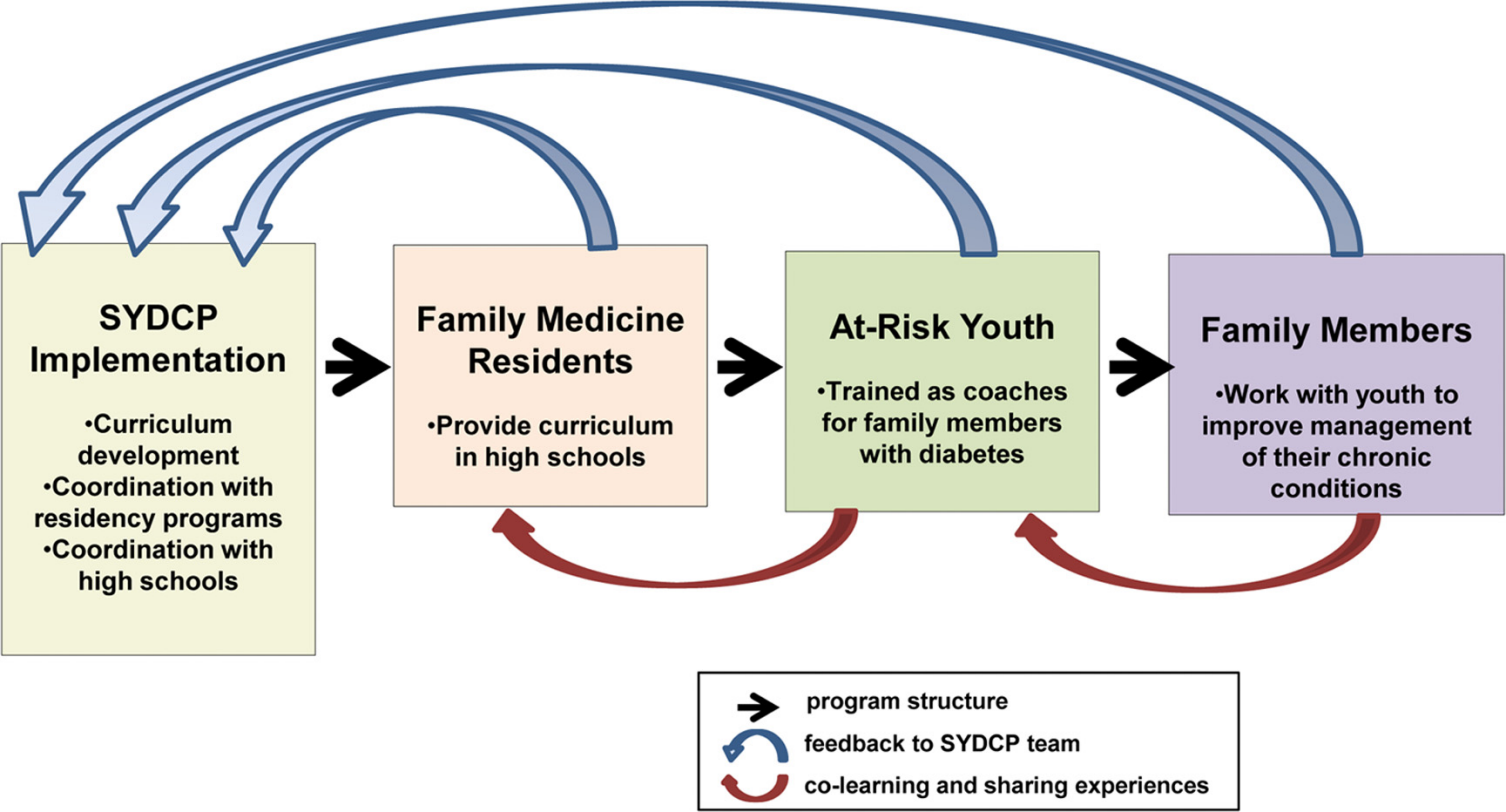
Structure, Feedback, and Co-Learning; Stanford Youth Diabetes Coaches Program, 2012–2015. Black arrows represent the structural implementation of the program; blue arrows represent program feedback shaping program development; and red arrows represent co-learning and sharing of experiences which occurs concurrently with program implementation.

The SYDCP is a “train the trainer program” in which Family Medicine residents train high school students to train their family members. The tightly scripted curriculum incorporates evidence- based approaches to chronic disease management[6], as well as feedback from Family Medicine resident instructors, high school student coaches, and the family members being coached. As summarized in Fig 1, the SYDCP team supports residency programs and high schools to develop partnerships and implement the program. Family Medicine residents go to the partner high schools and present the program curriculum once a week for eight weeks. The at-risk high school students attend the program classes where they are trained in coaching skills as well as basic diabetes and health knowledge. The student coaches then meet weekly outside of school with a family member with diabetes to complete a coaching assignment. The coaching assignments are structured such that the family members share their experiences and challenges managing their chronic illnesses with their student coaches. In turn, as part of structured check-ins and discussions, the student coaches share these realities with the Family Medicine residents in class. Program impact on student coaches is evaluated using pre- and posttest surveys.

### Sample and Setting

After the pilot study (2011–12)[4], a total of 10 high schools and one summer camp in the US and Canada and five Family Medicine residency programs were selected to participate in the program. From 2012–2015, physicians or other health providers implemented the SYDCP with racial and ethnic minority students from low-income communities in the US and Canada (Atlanta, GA; Redwood City, CA; Vallejo, CA; East Palo Alto, CA; San Jose, CA; Wilmington, DE; Ypsilanti, MI; and Kainai Blood Tribe Reserve, Canada).

Students were eligible to participate if they were in grades 9 through 12 (approximate ages 14–18). School and camp administrators determined whether the program would be voluntary or mandatory for participating students. In three schools, participation was voluntary, and students were recruited through advertisements posted in the schools. In seven schools and the summer camp, participation was mandatory and incorporated into students’ schedules. All participating students were asked to complete pre- and posttest surveys. In addition, in six participating schools, we obtained pre- and posttest surveys for 96 students not participating in the intervention to be used as a comparison group. The institutional review board at Stanford University approved the study. In the academic year 2012–2013, written informed consent was obtained from a parent or guardian of each participant; and written assent was obtained from each participant. In June, 2013, the institutional review board at Stanford deemed that the involvement of human subjects in our work was in a category that is exempt from the regulations at 45 CFR 46 or 21 CFR 56. The review board waived the need for written informed consent from the participants (including parents or guardians) after June 2013.

### Implementation Process

The process of initiating the SYDCP for Family Medicine residents and other health providers varied by site. Most first learned about the program at national meetings, through internet searches, and through word of mouth. For example, in Atlanta, one Family Medicine resident learned about the SYDCP and brought the information to her program director who made the opportunity available to all residents. In Vallejo, Family Medicine physicians who wanted to partner with a local underserved school asked to use the SYDCP as its complete curriculum would allow them to get started right away; in San Jose, the residency program community health director asked to utilize the SYDCP because he wanted his residents to participate in school outreach during their required community health rotation. In Wilmington, directors of a summer camp aimed at improving the health of at-risk youth and specifically targeting diabetes prevention wanted to incorporate the SYDCP so that Family Medicine residents visiting the camp could have a focused curriculum to provide the youth; in Ypsilanti, a Family physician who runs a school based health center asked to use the program to allow rotating Family Medicine residents to connect regularly with students in the classroom environment; and in the Kainai Blood Tribe Reserve, a community health nurse who was seeking an opportunity to combat the 50% diabetes rate in the community asked to use the SYDCP.

Although initial interest varied by site, functional implementation was consistent across all sites. One requirement of the health providers partnering with the SYDCP research team was that they conduct the program with strict adherence to SYDCP guidelines and specifications as described verbally and in written form by a member of the research team. At each site, the practical implementation of the program curriculum was exactly the same, as each site utilized the pre-made power point slides, instructor’s guides, and coaching assignments and held class once a week for an hour for eight consecutive weeks. Aside from the site in Canada needing to adapt the curriculum to show guidelines using the metric system; some sites needing to take a one week break mid-implementation due to mandatory school breaks; and the unavoidable differences in teaching styles of instructors, variability among students, and different site facilities, each site implemented the program the same way.

### Intervention

At each site, physicians or other health providers taught one-hour classes once a week for eight weeks using standardized web-based program curriculum focused on health knowledge, communication, problem-solving, and self-management skills. At all sites, Family Medicine residents taught the classes except in a few cases where Family Medicine residents were not available or where other health providers supplemented the teaching of the classes. Specifically, in the Kainai Blood Tribe Reserve, a community health nurse and registered dietitian taught the classes together because no Family Medicine residents were available; in Vallejo, Family Medicine physicians (not residents) taught the classes as they were piloting the work before starting a Family Medicine Residency Program where they would have residents teach the classes; in Wilmington, a dietitian taught together with Family Medicine residents; and in Redwood City, a Family Medicine physician and a school nurse took turns teaching the classes in order to provide the program to an at-risk group of students despite not having Family Medicine residents available. A detailed description of program curriculum can be found in the article describing the pilot implementation of the SYDCP.[4] Each participating student coached one family member with diabetes. Students who could not find family members with diabetes to coach were allowed to coach community members, friends, or peers. Additionally, students were allowed to coach individuals with pre-diabetes, other chronic illnesses, or simply those with a desire to improve their health. For simplicity, we refer to the individuals who were coached by students as family members with diabetes. Printed program materials were available in Spanish and English. Adaptations were made for the Canadian student coaches to be consistent with Canadian metrics.

**Fig 2:**
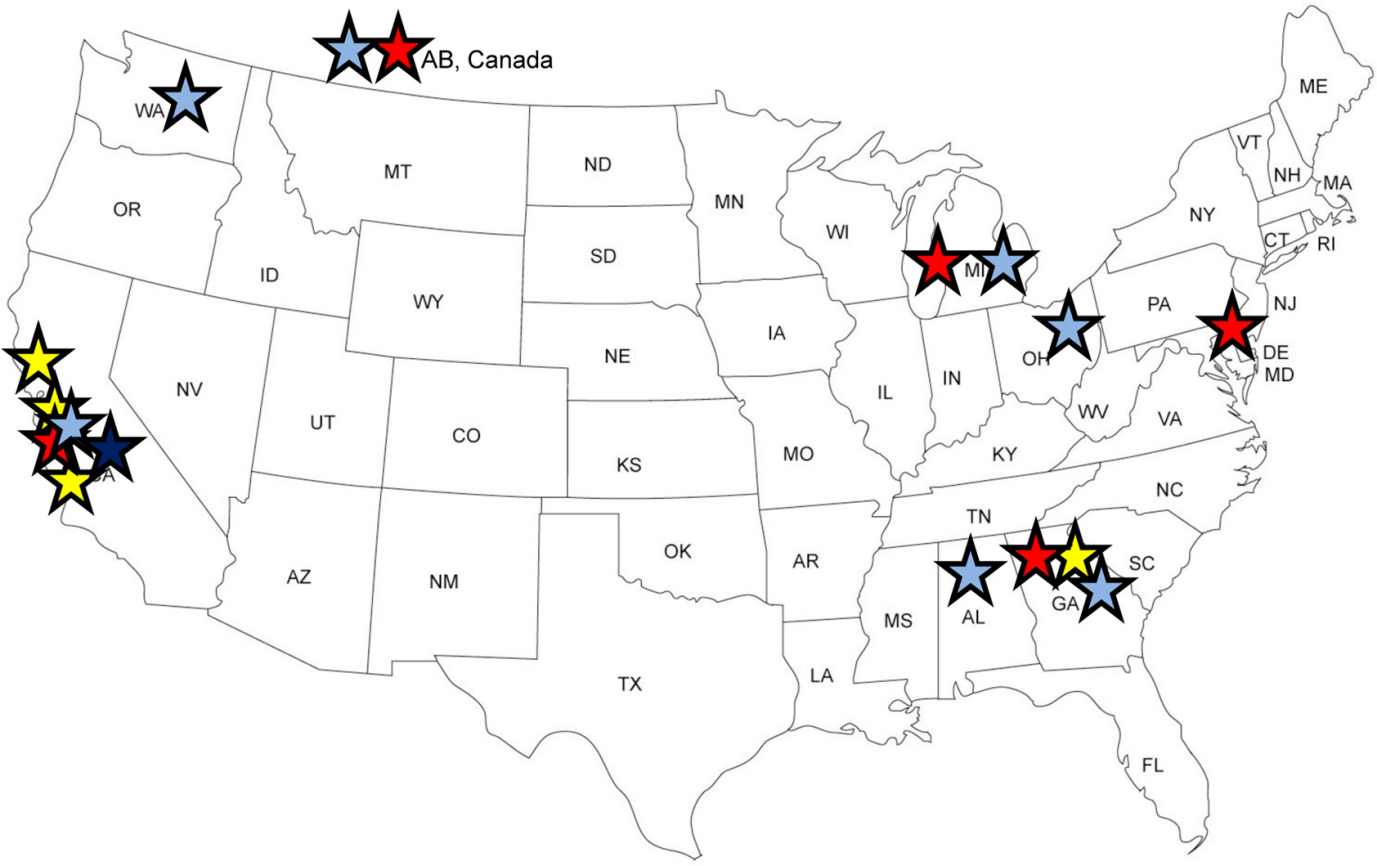
Implementation Sites By Year and Level of Curriculum Development, Stanford Youth Diabetes Coaches Program, 2012–2015. Yellow stars represent implementation of the original power point based curriculum in 2012–2013 with 3 residency programs at 8 high schools; red stars represent implementation of improved curriculum with the addition of various interactive elements with 3 residency programs, 3 high schools, and 1 summer camp in 2014–15; the dark blue star represents the pilot implementation of the technologically enhanced curriculum with 1 residency program and 1 high school in 2015; and light blue stars represent planned multi-site implementation of the technologically enhanced curriculum with 6 residency programs and 8 high schools. Reprinted from http://www.freeusandworldmaps.com/ under a CC BY license, with permission from Bruce Jones Design Inc., original copyright 2009.

As summarized in Fig 2, from 2012–2015, the SYDCP implementation at remote sites varied by level of curriculum development. The first phase of remote implementation of the SYDCP occurred in Fall 2012 and Spring 2013 at eight school sites located in Atlanta, GA; Redwood City, CA; San Jose, CA; and Vallejo, CA. Family Medicine residents from three residency programs implemented the SYDCP using the original curriculum. Based on qualitative feedback received from student coaches, Family Medicine residents, and high school teachers, as well as quantitative program evaluation data, the SYDCP team enhanced the curriculum to include more hands-on activities and discussions, and promote interaction between instructors and students. In Summer 2014, Fall 2014 and Spring 2015, enhanced curriculum was offered at three school sites located in Kainai Blood Tribe Reserve, Canada; San Jose, CA; and Ypsilanti, MI; and one summer camp located in Wilmington, DE. Based on additional feedback from this group, the SYDCP team further updated the curriculum with technological improvements including embedded short video segments, text reminders and encouragement; and an email program promoting behavior change through habit development. This technologically enhanced curriculum was offered to one small group of students as a pilot in the Spring of 2015; and will be offered in 2015–2016 at five additional school sites in Birmingham, AB; Cincinnati, OH; San Jose, CA; and Seattle, WA.

### Measures and Analysis

#### Outcome measures

Student coaches and a group of non-participating students completed pre- and posttest surveys which included questions on knowledge, psychosocial assets, and health behaviors, as well as open-ended feedback questions. Diabetes related knowledge was measured using questions from the validated Michigan Diabetes Research and Training Center’s Brief Diabetes Knowledge Test [7] and the Spoken Knowledge in Low Literacy in Diabetes scale [8], as well as knowledge questions developed by the SYDCP team derived directly from program curriculum. We measured psychosocial assets including self worth, sense of belonging, and resilience using scales adapted from the validated California Healthy Kids Survey [9] and the Search Institute scales.[10,11] Health behavior questions included questions about fruit and vegetable consumption and were adapted from Physical Health and Nutrition Module Supplemental Series 2 of the validated California Healthy Kids Survey which asks student participants to note how many times they ate fruit or vegetables in the last 24 hours. In addition, posttest surveys for participants included questions about intention to change health behaviors or lifestyle habits associated with program participation, as well as open-ended questions about what participants would change about the program and what they liked best. More details about the specifics of each measure used can be found in the article describing the pilot implementation of the SYDCP.[4] One small group of students participated in the new technologically enhanced curriculum and completed an additional new set of questions to elicit feedback about the technological enhancements.

#### Quantitative data analysis

Participant students with complete pre- and posttest data were included in the analysis. We used SPSS 23 and STATA 13 for the data merge and analysis. To measure improvement in knowledge levels, behavior change and psychosocial assets, we compared mean difference in pre- and posttest scores of participating student coaches using *t* tests to determine p values for level of significance. The data was divided by year, setting, and curriculum type into four cohorts: Fall 2012- Spring 2013 with original curriculum, 2014 summer camp, Fall 2014- Spring 2015 with revised curriculum, and Spring 2015 pilot with technologically enhanced revised curriculum. In this paper, we refer to the groups as 2012–13, 2014 summer camp, 2014–15 and 2015. Of the 13 students who enrolled in the Spring 2015 pilot, seven completed both pre- and posttests. Although that cohort (n = 7) is included in our results, the sample size is too small to statistically assess change and was therefore not considered for quantitative analysis. Feedback data was analyzed, however, from all participants in this small cohort who completed a posttest in which they provided responses about the technological enhancements (embedded video, text messages, and email encouragement for behavior change) (n = 10).

#### Qualitative data analysis

For the qualitative data analysis, we applied open and axial coding methods[12,13] to determine themes and categories during analysis of three open-ended survey questions. The open-ended questions were initially read by two of the authors (L.G., A.S.) who independently determined themes based on repetition of responses and coded responses accordingly. Subsequent to initial coding, an additional double-blind peer review was conducted by another author (E.R.) to independently verify the coding categories and minimize bias. In cases of discrepancies in coding, the 3 investigators discussed and reached consensus.

## Results

In total, 311 high school students enrolled in the SYDCP as program participants from Fall 2012-Spring 2015 and completed pre-test questionnaires. 272 participating high school students completed posttests. Of these 272 participants, 216 students completed both pre-and post tests matched by unique identification codes. Based on direct verbal and written communications with high school staff who facilitate the SYDCP at all sites, explanation of the discrepancy between the numbers of students enrolling (taking the pre-test), finishing (taking the posttest), and completing the full program (taking both the pre- and posttest) stems from the fact that many of the student participants have multiple life-stressors that prevent them from attending school each day. Often students who begin the program are unable to complete it due to such factors as: poor school attendance; other commitments such as caring for elderly family members or younger siblings; or schedule conflicts with other activities. For similar reasons, students often join the program after the first session and are not offered the opportunity to complete a pre-test. We also collected pre- and posttest survey data for 96 nonparticipating students in six of the schools to serve as a comparison group.

Although the demographic characteristics were similar between participating students and the comparison group, nonparticipants did not demonstrate improvements in any of the measures analyzed (data not shown). These results were similar to those reported in our pilot study which demonstrated statistically significant improvements in the areas of knowledge, belonging, and worth in participants versus nonparticipants.[4]

As summarized in Table 1, the majority of student participants identified themselves as African American (49%) and Hispanic or Latino (24%). Participants were more likely to be female (66.2%), less than half were living at home with two parents (42.6%), and approximately one in seven mostly spoke a language other than English at home (13.4%).

**Table 1:** Demographics of Stanford Youth Diabetes Coaches Program participants (n = 216); California, Canada, Delaware, Georgia, and Michigan, 2012–2015.

Ethnicity	2012–13^a^ (N = 128)	2014^b^ Summer Camp (N = 37)	2014–15^c^ (N = 44)	2015^d^ (N = 7)	Total (N = 216)
African American or Black	73 (57%)	26 (70.3%)	7 (15.9%)	0	106 (49%)
Asian or Asian American	12 (9.4%)	0	16 (36.3%)	4 (57.1%)	32 (14.8%)
Hispanic or Latino/a	34 (26.6%)	7 (18.9%)	9 (20.4%)	2 (28.6%)	52 (24%)
White or Caucasian	10 (7.8%)	6 (16.2%)	4 (9%)	2 (28.6%)	22 (10.2%)
Other^e^	17 (13.3%)	13 (35.1%)	11 (24.9%)	1 (14.3%)	42 (19.4%)
**Total**	146	52	47	9	254*
**Gender** (Female)	95 (74.2%)	18 (48.6%)	25 (56.8%)	5 (71.4%)	143 (66.2%)
Living with both parents	61 (47.7%)	6 (16.2%)	20 (45.5%)	5 (71.4%)	92 (42.6%)
Mostly speak language other than English at home	15 (11.7%)	3 (8.1%)	9 (20.5%)	2 (28.6%)	29 (13.4%)

* Some students marked more than one ethnicity

^a^Multisite schools in California and Georgia

^b^Summer Camp, Delaware

^c^Multisite schools in California, Canada and Michigan

^d^Single School in California with enhanced curriculum

^e^Other includes: 1. American Indian or Alaska Native 2. Native Hawaiian or Pacific Islander 3. Other ethnicity

### Quantitative Results

As described in Table 2, student coaches improved from pre- to posttest significantly on knowledge (p<0.005 in 2012–13, 2014 camp, and 2014–15); worth (p<0.1 in 2014–15); problem solving (p<0.005 in 2014 camp and p<0.1 in 2014–15); and self-efficacy (p<0.05 in 2014 camp). Participants enrolled in a summer camp in 2014 also showed significant improvement in consumption of fruits (p<0.005) and vegetables (p<0.05). There was no significant increase in a sense of school belonging associated with the intervention (data not shown). Additionally, 82.3% of student coaches reported considering making a behavior change to improve their own health as a result of program participation.

**Table 2:** Mean Test Scores, Differences and Percent Differences by Year and Curriculum Development (n = 216); Stanford Youth Diabetes Coaches Program; California, Canada, Delaware, Georgia, and Michigan, 2012–2015.

	Original Curriculum	Revised Curriculum	Revised Curriculum	Revised and technologically enhanced curriculum
	2012–13 (N = 128)	2014 Summer Camp (N = 37)	2014–15 (N = 44)	2015 (N = 7)
**Pretest**	**Mean**	**Mean**	**Mean**	**Mean**
Knowledge	9.23	8.54	8.57	10.86
Behavior^f^: Eat vegetable	2.80	2.65	2.68	1.86
Eat fruit	3.06	3.05	2.80	3.43
Worth 6–9^g^	12.34	12.62	12.11	13.43
Resilience: Problem solving	NA	5.46	5.41	6.29
Self Efficacy	NA	13.19	NR	NR
**Posttest**	**Mean**	**Mean**	**Mean**	**Mean**
Knowledge	13.78	12.43	13.11	15.43
Behavior: Eat vegetable	3.02	3.51	2.34	2.86
Eat fruit	2.98	3.78	2.82	3.71
Worth 6–9	12.53	13.14	12.68	14.29
Resilience: Problem solving	NA	6.51	6.02	6.71
Self Efficacy	NA	14.05	NR	NR
**Mean Difference**	**Post-Pre test**	**Post-Pre test**	**Post-Pre test**	**Post-Pre test**
Knowledge	4.547***	3.892***	4.543***	4.571
Behavior: Eat vegetable	.220	.865**	-.341	1.000
Eat fruit	-.079	.730***	.023	.286
Worth 6–9	.189	.514	.568*	.857
Resilience: Problem solving	NA	1.054***	.614*	.429
Self Efficacy	NA	.865**	NR	NR
**Percent change**				
Knowledge	49.2%	45%	53%	42%
Behavior: Eat vegetable	7.8%	32.6%	-12.7%	53.7%
Eat fruit	-2.5%	24%	0.8%	8.3%
Worth 6–9	1.5%	4.07%	4.6%	6.3%
Resilience: Problem solving	NA	19.02%	11.3%	6.8%
Self Efficacy	NA	6.5%	NS	NS

^f^ how many times vegetable or fruit was eaten in the last 24 hours

^g^Important to give back to family; Important to give back to neighborhood; Get adult to see my point of view; Enjoy influencing actions of others

NA = Not assessed, NR = Not reported, NS = Not significant

P values calculated per T test from mean difference

* p < .1

**p < .05

*** p < .005

Additionally, we further categorized the data based on whether the program was offered in a mandatory or elective setting. Pre-test scores for knowledge and worth for student coaches in elective programs (n = 50) were consistently higher (15% higher for knowledge; 5% higher for worth) than pre-test scores for students in mandatory programs (n = 166). When pre and post-test scores were compared, student coaches enrolled in mandatory programs demonstrated significant improvement in knowledge levels, worth, problem solving and self-efficacy. Student coaches in elective programs showed significant improvement in pre and post test-scores in knowledge levels, but not in worth, problem solving, or self-efficacy.

### Qualitative Results

**Fig 3:**
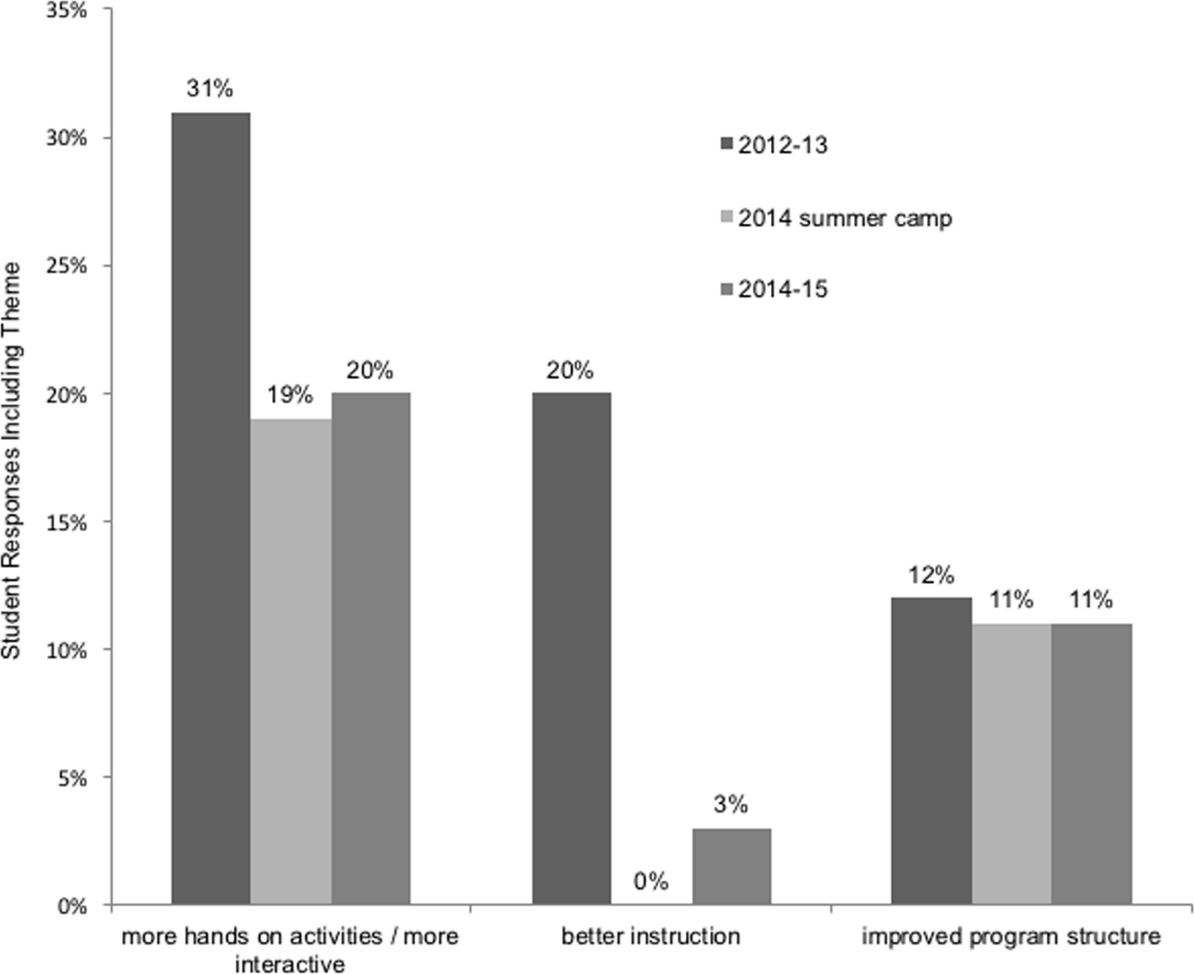
Percentage of Student Coaches’ Responses Including Major Program Improvement Themes by Year and Curriculum Development (n = 195); Stanford Youth Diabetes Coaches Program; California, Canada, Delaware, Georgia, and Michigan, 2012–2015. The 2015 pilot group is not included in this analysis due to its small size.

When asked what they would like to change or make better about the program, student coaches answered with three main themes: making the program more interactive or including more hands-on activities; improving instruction, and improving program structure. As shown in Fig 3, the percentage of students suggesting these changes decreased as the years progressed. The general trend for these responses is that fewer students had suggestions for improvements as the curriculum was enhanced.

Several themes emerged in the analysis of the qualitative feedback from student coaches answering the question “What did you like best about the program?” Table 3 describes the percentage of student participants who mentioned one of the following themes in their responses: knowledge gain associated with the program (37%), pride in being able to help family members with diabetes (14%), improved relationships and connectedness with family members (8%), acknowledgment of the usefulness and relevance of the program (20%), appreciation for the physician instructors (18%), and program’s role in improving lifestyle (12%).

**Table 3:** Sample Responses by Theme from Student Coaches’ Answers to Questions: “What did you like BEST about this program?” and “Please write down the [behavior] changes you have considered making.” (n = 201); Stanford Youth Diabetes Coaches Program; California, Canada, Delaware, Georgia, and Michigan, 2012–2015.

“What did you like BEST about this program?”	Number of student responses including this theme (%)	Sample Student Coach Responses
Learning more about diabetes and health	75 (37.3%)	I was able to better understand the struggles that people with diabetes go through, and learned a lot about how to maintain good health from my grandmother who I was coaching.
Being able to help someone	28 (13.9%)	I like that I got to learn new things that I can use to help myself and to help my mom and best friend. Having to coach someone and teach them what you've learned and help them gain a better life.
Improved relationship and connectedness with family member	16 (7.9%)	Us students got to interact with a team member** and slowly build up a trusting relationship. Learning how to listen to a team member** really encouraged and motivated them to work towards their goal.
Relevant and useful curriculum	40 (19.9%)	I liked the discussions on what we were doing as coaches with the team members**.The homework and class work are not hard but very simple and useful.
Appreciation for having doctors teach classes	37 (18.4%)	I like[d] having doctors come in and do hands on activities with us. I liked how friendly the doctors were and how seriously they answered our questions.
Program helped improve lifestyle for my family and/or me	24 (11.9%)	I liked doing the action plans because they helped both me and my team member** to achieve a goal. My mother and I learned a lot about our lifestyle and how we could change some habits for the better from the program.
Total responses	220 (109.4%*)	
**Have you considered making any changes in diet, physical activity, or some other behavior after taking the Stanford Youth Diabetes Coaches class?**	**Yes (82.3%)**	
**“Please write down the changes you have considered making.”**	**Number** (**%**)	
Improve diet	120 (59.7%)	I have eaten less sugary stuff because I realized the causes of diabetes and I, myself, was in the prestage of getting diabetes and now I can control it. I haven't had a lot of candy or anything sweet other than a cup of fruit juice every other day.
Increase physical activity	79 (39.3%)	Jogging to and from the train station before and after school in order to get a little more physical activity.
Weight reduction	5 (2.5%)	lose weight, get fitted[r]
Stress reduction/improve sleep	4 (2.0%)	To cut out the non healthy foods, get a good amount of rest each night, and to not stress so much and time management.
Total responses	208 (103.5%*)	

*Values do not always add to 100% because some respondents’ answers included more than one theme and some respondents did not respond to the question.

**SYDCP curriculum uses the term “team member” to refer to the person being coached and reinforce the collaborative nature of health coaching.

When asked if they had considered making any changes in diet, physical activity, or some other behavior after program participation, 82.3% of student coaches responded affirmatively. When asked to describe the behavior changes, 60% cited improving their diets, 39% cited increasing physical activity, 3% cited weight reductions, and 2% cited reducing stress or improving sleep.

### Feedback from Pilot for Technologically Enhanced Curriculum

Of those who received the technologically enhanced curriculum, all students reported that the videos were useful in becoming better diabetes coaches; that the emails increased their confidence in creating good habits for the future; and that receiving texts was useful and helped remind them about their coaching work. Ninety-two percent reported the in-class videos were interesting; and 83% reported sharing the email tips with the family members they were coaching.

## Discussion

This study demonstrates that the SYDCP can be reproduced in a variety of settings in North America and has the potential to equip at-risk youth with knowledge and skills to enable them to become engaged in the health of their family members and empowered to improve their own health behaviors. All cohorts displayed significant improvements in diabetes and health related knowledge, confirming that the program structure reliably and effectively boosts diabetes and health related knowledge.

Results from early dissemination of the program (2012–13) demonstrated, however, that 1) program participation was not improving student coaches’ psychosocial assets; and 2) student coaches wanted a more interactive program. These results were used to revise the SYDCP curriculum to emphasize discussion and interaction between physicians and students. Results after the curriculum revision show that incorporating these interactive elements into the program increases the potential to improve psychosocial assets such as self-efficacy to solve problems and work with others, while simultaneously improving student coaches’ perception of the program. Improvements in psychosocial assets are particularly important in empowering youth to improve their health behaviors and reduce risk-taking behaviors as emphasized in Healthy People 2020[14] and demonstrated in the peer-reviewed literature.[15–17]

The results also indicate that the SYDCP has potential to improve psychosocial assets among high school students who are required to participate in the program, as well or even more than those who elect to participate. One likely explanation for student coaches in mandatory settings showing more improvement than those in elective settings may stem from the baseline differences between student coaches in elective versus mandatory settings. On the whole, student coaches who participated in elective programs have higher pre-test scores for knowledge and assets than those in mandatory programs (data not shown), and thus, less room for improvement.

Often it is easier for schools with few resources to have SYDCP sessions taught during the school day in a mandatory setting during an already existing class because school staff is not available to organize and manage elective sessions. The observation that students participating in mandatory compared to elective settings show more improvement will help the SYDCP research team assure these sites that mandatory classes are effective for their students. However, the research team will also encourage elective classes because it is the experience of the research team that in elective classes, the students’ desire to participate creates a more engaged and peer-supportive dynamic.

The SYDCP does not appear to have a consistent impact on health behavior change of the participant youth based on the quantitative behavior change questions utilized in the survey. It may be that in focusing on health coaching, the SYDCP curriculum does not sufficiently encourage youth behavior change. Alternatively, it may be that the quantitative behavior change questions do not capture the changes the youth are making, as the vast majority of student coaches reported making a behavior change to improve their health secondary to program participation. The significant improvements in consumption of fruits and vegetables seen in summer camp students (Table 2) could have been fostered by the added camp focus on healthy food choices.

Lastly, use of short embedded video to teach basic content quickly in each class allowed more time for meaningful interaction between student coaches and physicians in class. Text messages and emails have been shown to be an effective tool for engaging youth in positive health behaviors[18,19] and increasing a sense of connectedness with health programs.[20] Additionally, behavior change through habit formation has been shown to be effective in youth.[21] For these reasons, our future work will include these enhancements, and we will evaluate their impact on a larger scale.

The qualitative data provides a window into the value of the program for the student coaches, the shortcomings of the program, and the influence of the program on behavior change.

Themes extracted from the question “What would you like to change or make better about this program?” were valuable in the iterative development of the program. In the final years of implementation there were fewer students suggesting program improvements compared to earlier years, indicating that the modifications made to the curriculum at subsequent stages of implementation played a role in addressing these program shortcomings.

The themes extracted from the open-ended survey question “What did you like best about the program?” are consistent with themes extracted from interviews with participants in the SYDCP pilot study[4] and are informative in several ways. The large number of students who describe learning more about diabetes as the best part of the program (Table 3) corroborates our quantitative data demonstrating a significant increase in diabetes and health knowledge after the program. It is encouraging that many student coaches noted their favorite part of the program was either being able to help someone or the way the program enhanced their relationships with family members (Table 3). These qualitative data suggest program participation has the potential to improve family relationships which is a key predictive feature of student’s future health behavior as adults.[22] A number of student coaches also cited the best part of the program as how it encouraged intra-family lifestyle changes that promote health (Table 3). In this way, program participation empowered student coaches to be agents of change in their families. Lastly, a large number of student coaches noted that having the doctors teach the classes is what they liked best; and many noted how helpful and friendly the doctors were (Table 3). Given many ethnic minority groups’ historically justified and continued mistrust in the healthcare system[23], it is possible that partnership programs such as the SYDCP may work to rebuild trust.

The qualitative data describing the behavior change plans of the student coaches suggest that program participation may play an important role in encouraging at-risk youth to adopt healthy behaviors (Table 3). The qualitative responses describing how student coaches connected with and helped family members suggest that program participation may encourage family members of student coaches to improve their health behaviors as well. The challenge of facilitating behavior change among adolescents is well documented[24], and many studies conclude that the approach to behavior change must include family participation in order to be successful.[25] The SYDCP promotes behavior change and integrates the participation of family in a unique way in that it empowers the student coaches to be the agents of change within the family.

In review of the literature, a number of programs aim to reduce risk factors for and/or prevent the onset of diabetes in ethnic minority youth. One program found that using a community based participatory research approach was an effective strategy to adapt the highly successful National Diabetes Prevention Program[26] for adults to be useful for ethnic minority youth.[27] A variety of programs targeting ethnic minority youth have had positive results including finding that when youth make a public commitment to improved health behaviors, obesity rates decline[28]; youth respond with decreases in BMI and increases in self-reported walking[29]; obese children decrease BMI and improve insulin sensitivity and glucose tolerance[30]; and youth show significant decreases in capillary glucose levels and percent body fat, as well as significant increases in physical activity capacity.[31] To the best of the authors’ knowledge, however, there are no such nationally disseminated programs for youth; no such programs aimed at empowering at-risk youth to become health coaches for family members with chronic illness; and no such programs facilitating a partnership between the medical community and underserved schools. The available studies demonstrate that youth interventions can significantly reduce risk factors for developing diabetes, but in each of these studies, there is no mention of a plan for scalability, a plan to provide these interventions to more at-risk youth. The SYDCP system of using medical residents as instructors leverages resources in the community to provide a successful intervention on a larger scale with minimal cost.

### Challenges

The logistics of partnerships between underserved schools and residency programs can present challenges in communication and scheduling. It is the experience of our research team that underserved schools are frequently understaffed and overburdened, and as a result, lapses in communication about the SYDCP have occurred. Additionally, unforeseen occurrences such as inclement weather or special programs contribute to challenges of scheduling. Furthermore, it is frequently difficult to obtain data from all participating high school students. The research team is frequently unable to evaluate the impact of the program because many students to not complete either the pre or the post-test due to challenges described above in the results section. Additionally, because school staff have multiple responsibilities and rarely have assistance, staff usually offer the pre- and posttests at one time only and do not offer “make-ups.” Likewise, if the designated SYDCP school site staff member is not available, pre- and/or posttests may not be offered at all. For example, one group of 12^th^ grade student participants completed pre-tests, attended all eight program sessions, but did not complete posttests because the school site staff member was ill and not able to provide the posttest before the seniors finished their shortened academic year.

### Study Limitations

This study was limited in that post participation evaluation only occurred immediately after the eight-week program and therefore could not assess duration of program benefits. Additionally, apart from student coaches’ qualitative reporting of family member benefit and phone interviews during the pilot testing phase of the project, our research team did not assess program impact for the family members being coached. Evaluation was limited because it included self-report measures to assess changes in health behaviors which are known to be less accurate than objective measures; and because the sample size was so small for the evaluation of the technologically enhanced curriculum pilot, it is difficult to draw any formal conclusions. Lastly, there is inherent variability of program administration based on differences in instructors, students, and program sites. Although we could analyze program impact for specific settings, instructors, and students, here we are combining sites for much of the analysis, and therefore representing only an overall median outcome.

### Next Steps

Currently, the SYDCP is only an eight-week intervention. Our research team aims to encourage prolonged engagement with health promotion methods and the use of self-management skills beyond the eight weeks. By utilizing online resources and social media, as well as mobile technology such as text messages, the program could allow student coaches, family members, and physician instructors to stay connected, share resources, and gain access to current health information. Concordantly, our team plans to evaluate the student coaches at several time points after participation to assess the duration of program benefits. Additionally, our team will assess the program impact on family members being coached in the program.

## Conclusion

The SYDCP is an effective, scalable intervention that can be reliably reproduced at sites around North America. This program can increase health knowledge and some psychosocial assets of at-risk students and holds promise to empower student coaches with health literacy and encourage them to adopt healthy behaviors. In concert with training primary care physicians to better partner with their patients, the SYDCP has the potential to equip at-risk populations to become engaged in healthcare and empowered to improve their own health. This program illustrates the potential of partnerships between schools, physicians, and families, and the benefit of an iterative process of program development and dissemination in order to meet the needs of all partners. The SYDCP effectively leverages the inherent altruism of teens to help their family members and the aspirational goals of physicians in training to help communities realize a diabetes education and prevention system that is accessible, sustainable and reproducible—even for under resourced communities.
